# Landscape of Secondary Findings in Chinese Population: A Practice of ACMG SF v3.0 List

**DOI:** 10.3390/jpm12091503

**Published:** 2022-09-14

**Authors:** Yingzhao Huang, Bowen Liu, Jile Shi, Sen Zhao, Kexin Xu, Liying Sun, Na Chen, Wen Tian, Jianguo Zhang, Nan Wu

**Affiliations:** 1 Department of Orthopedic Surgery, State Key Laboratory of Complex Severe and Rare Diseases, Peking Union Medical College Hospital, Peking Union Medical College and Chinese Academy of Medical Sciences, Beijing 100730, China; 2 Beijing Key Laboratory for Genetic Research of Skeletal Deformity, Beijing 100730, China; 3 Key Laboratory of Big Data for Spinal Deformities, Chinese Academy of Medical Sciences, Beijing 100730, China; 4 Department of Hand Surgery, Beijing Jishuitan Hospital, Beijing 100035, China; 5 Department of Obstetrics and Gynecology, Peking Union Medical College and Chinese Academy of Medical Sciences, Beijing 100730, China

**Keywords:** clinical exome sequencing, secondary finding, clinical genetics, rare disorders

## Abstract

Clinical exome sequencing (CES) has shown great utility in the diagnosis of Mendelian disorders. CES can unravel secondary findings (SFs) unrelated to the primary diagnosis but with potential health implications. The American College of Medical Genetics and Genomics (ACMG) has published a guideline for reporting secondary findings and recently updated an ACMG SF v3.0 list comprising 73 genes. Several studies have been performed to explore the prevalence of SFs. However, the data were limited in the Chinese population. In this study, we evaluated the genetic data of 2987 individuals from the Deciphering Disorders Involving Scoliosis and COmorbidities (DISCO) study group in accordance with the ACMG SF v3.0 list. The detected variants were evaluated using the ACMG classification guidelines, HGMD, and ClinVar database. Totally, 157 (157/2987, 5.3%) individuals had reportable variants within genes associated with cancer, cardiovascular, metabolic, and miscellaneous phenotypes. We identified 63 known pathogenic (KP) variants in 72 individuals (72/2987, 2.4%) and 96 expected pathogenic (EP) variants in 105 individuals (3.5%). Forty-five individuals carried SFs in v3.0 newly added genes, which accounted for 1.5% of our cohort. Our findings could contribute to existing knowledge of secondary findings in different ethnicities and indicate the necessity for clinicians to update the SFs gene list.

## 1. Introduction

In the last two decades, a rapid advance in molecular technology has dramatically promoted the development of exome/genome sequencing, which makes the genomic data of patients widely accessible [1]. The identification of pathogenic mutations in specific genomic regions assists the diagnosis of genetic disorders, and further guides disease prevention and precise management. The variants in disease-causing genes unrelated to the patient’s primary concern but posing a potential medical value are also extracted, which are termed as secondary findings (SFs) [2]. SFs variants have been related to severe genetic disorders, some of which are available for efficacious interventions [3]. Although those variants are usually unrelated to the primary purpose of clinical exome/genome sequencing, it is still necessary to report those secondary findings because of their clinical significance in the prediction, prevention, and early intervention in the progression of life-threatening genetic diseases [3,4].

In 2013, the American College of Medical Genetics and Genomics (ACMG) published the first recommendation on responsible management of SFs when patients undergo exome or genome sequencing [3]. The known pathogenic (KP) and expected pathogenic (EP) variants in 56 genes should be reported even though they are unrelated to the primary testing indication. The second version of the recommendation was subsequently proposed four years later, with four new genes (*ATP7B*, *BMPR1A*, *SMAD4*, *OTC*) added and *MYLK* removed. Recently, the ACMG published the latest version of the medically actionable gene list in 2021 [4]. A total of 14 genes were newly introduced into the revised list, indicating a dramatic development in human genetics and disease etiology over the past few years. According to those three versions of ACMG recommendations, several studies have reported the medically actionable variants in different cohorts consisting of distinct ethnicities, including Hong Kong Chinese, Lebanese, Osmanli, Korean, Thai, Qatari, etc., in the past few years [5,6,7,8,9,10]. Those reports made the prevalence and epidemiology of those SFs variants in different populations better accessible, and thus contributing to the existing knowledge of SFs variants and their clinical significance.

In this study, we screened for the secondary findings according to the recommendation of the ACMG SF v3.0 list in our heterogeneous cohort consisting of patients diagnosed with scoliosis, Mayer–Rokitansky–Küster–Hauser syndrome, short stature, congenital limb malformation, etc. This work provided insights into the epidemiological landscape of SFs in the Chinese population. Furthermore, we evaluated the accessible clinical phenotypes and family history of the SFs variant carriers, which might contribute to the comprehensive understanding of the clinical significance of secondary findings.

## 2. Materials and Methods

### 2.1. Patient Enrollment

**Figure 1:**
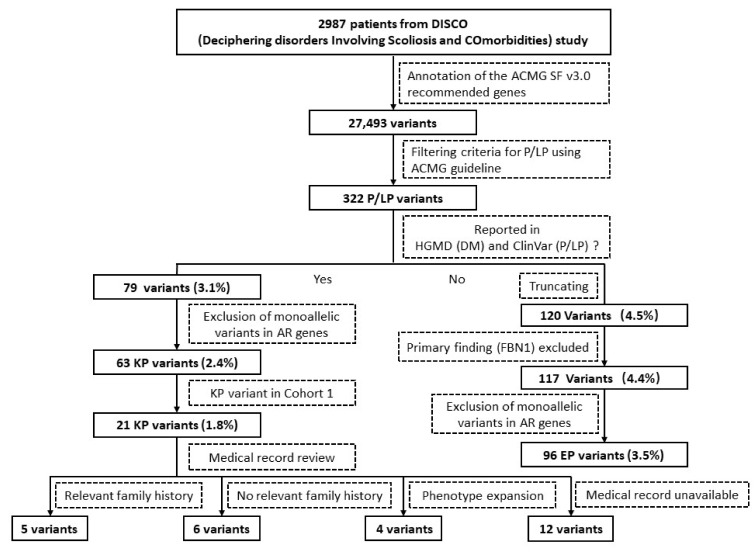
Schematic workflow of the secondary findings (SFs). All the percentages listed in the figure indicate the proportion of SFs variant carriers in our cohort.

Exome sequencing data of 2987 patients from the DISCO study was evaluated (Figure 1). Adequate informed consents of wide-ranging genetic studies were obtained from all patients or guardians of patients. Ethics approval for the study was obtained from the ethics committee at the Peking Union Medical College Hospital (JS-2364).

### 2.2. Sequencing and Variant Calling

All patients underwent the same procedure of DNA preparation from blood. ES was performed on peripheral blood DNA. Illumina paired-end libraries were prepared from DNA samples. All sequencing procedures were previously described [11,12,13,14]. The data were analyzed by an in-house-developed Peking Union Medical College Hospital Pipeline (PUMP) [12,14].

### 2.3. Variant Interpretation

Variant interpretation was limited to the 73 genes recommended by the American College of Medical Genetics (ACMG) in the SF v3.0 list [4]. The ACMG variant classification guideline was applied for all variants [15]. Variants that were classified to be pathogenic/likely pathogenic (P/LP) by InterVar [16] were defined as candidate variants. Candidate variants listed as P/LP in ClinVar (version 20210718) or DM (Disease Causing Mutation) in the Human Gene Mutation Database (HGMD) (version 202004) were categorized as KP (known pathogenic) variants. The rest of the candidate variants, which were previously unreported but could cause loss-of-function (Lof) of the gene (including nonsense, frameshift, and canonical splice-site variants) were categorized as EP (expected pathogenic) variants. For the *HFE* gene, only *HFE* p.Cys282Tyr was retained. Additionally, we evaluated the medical history of all patients with reportable variants and excluded 3 patients with Marfan’s syndrome carrying *FBN1* mutations as primary indication. Considering the design purpose of this reporting research, we did not perform in vivo or in vitro experiments to evaluate the biological impact of identified variants. However, functional validation of the newly discovered SFs variants remains to be performed to obtain more accurate frequency data in the future.

## 3. Results

### 3.1. Actionable EP and KP Variants

**Figure 2:**
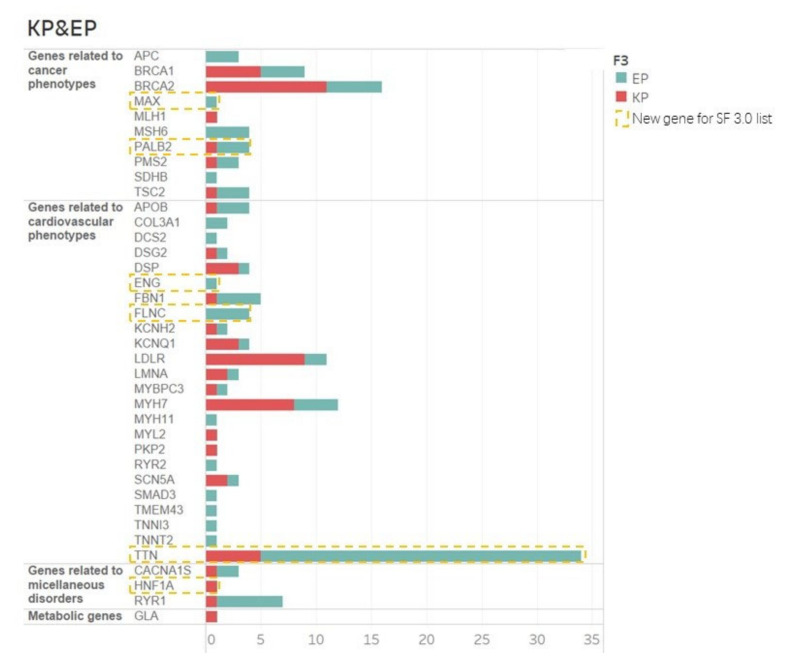
An overview of secondary findings (SFs) in this study.

According to the 2015 ACMG/AMP standards and guidelines for variant classification, 283 distinct variants were defined as reportable variants. Among these variants, 79 reportable variants in 92 individuals (3.1%; 92/2987) reported as P/LP in ClinVar or DM in HGMD were classified as KP variants, while the 120 remaining truncating reportable variants in 134 individuals (4.5%; 134/2987) were classified as EP variants (Supplementary Table S1). Subsequently, three patients with Marfan’s carrying FBN1 mutations as primary indication were excluded (Supplementary Table S1). According to the ACMG guidelines for reporting secondary findings, a single variant in genes associated with phenotypes inherited in a recessive fashion could not meet the threshold for reporting. Therefore, a total of 37 variants, including 16 KP variants and 21 EP variants, were also excluded from the reporting list, leaving 63 KP (in 72 individuals; 2.4%; 72/2987) and 97 EP variants (in 105 individuals; 3.5%; 105/2987) reported as secondary findings (Supplementary Table S1). A sum of 38 genes were associated with 17 autosomal-dominant diseases and 1 X-linked disease, which could be classified into four specific phenotype categories: cancer, cardiovascular, metabolism, and miscellaneous phenotypes, according to the ACMGF SF v3.0 list (Figure 2). All the individuals carrying those SFs variants have been contacted and informed of the genetic abnormalities and potential disease risk.

SFs variants related to cancer phenotypes were found in 54 individuals constituting 30.9% (54/175) of the individuals with SFs and 1.8% (54/2987) of our cohorts. Patients with KP variants accounted for 40.7% of them (22/54) while patients with EP variants accounted for 59.3% (32/54). The most frequent SFs gene was BRCA2 (16 variants in 19 individuals), followed by BRCA1 (9 variants in 9 individuals), both of which were associated with increased susceptibility to breast and ovarian cancer. [17,18]. We also identified medically actionable variants in eight other cancer-related SFs genes, including APC, MAX, MSH6, MLH1, PALB2, PMS2, SDHB, and TSC2 (Supplementary Table S1). 

The medically actionable variants associated with cardiovascular phenotypes were detected in 105 individuals representing 60.0% (105/175) of the individuals with SFs and 3.9% (105/2987) of our cohorts. Among those patients, 45.7% (48/105) of them carried KP variants while the others (54.3%; 57/105) carried EP variants. The variants of TTN, a new dilated-cardiomyopathy-related gene included in the SF v3.0 list, occupied the largest proportion of those SFs variants. We have identified 34 TTN variants in 35 individuals, accounting for 1.2% of our cohort (35/2987).

We also identified 12 variants in 4 genes related to miscellaneous phenotypes in 13 patients who represented 14.2% (13/175) of the individuals with SFs and 1.1% (12/2987) of our cohorts, including 3 KP variants (25%, 3/12) and 9 EP variants (75.0%, 9/12). Moreover, only one missense variant related to metabolism phenotypes was identified in our cohorts. The variant (GLA, c.888G > A) was labeled as a pathogenic variant in the ClinVar database and was carried by one patient. The reported carriers of these variants were diagnosed with Fabry disease, which follows an X-linked inheritance model. 

### 3.2. SF v 3.0 Updated Genes

Based on the updated SFs gene list (v3.0), a total of 14 new genes were newly included for SFs variants reporting. We have screened for the SFs variants of those genes in our cohorts and identified 7 KP variants (in 8 individuals; 0.3%; 8/2987) and 38 EP variants (in 38 individuals; 1.3%; 38/2987) in six genes, including TTN, ENG, FLNC, HNF1A, MAX, and PALB2 (Supplementary Table S1). The variants of SF v3.0 updated genes accounted for a large proportion (28.3%, 45/159) of the medically actionable variants. The new version of the SFs gene list has identified more SFs variants compared with the SFs v2.0 list (159 to 114) in our cohort. More carriers of pathogenic/likely pathogenic variants were hence informed of the potential disease risk in the future, indicating the necessity of clinicians to update the SFs gene list.

### 3.3. Phenotypes of KP Variant Carriers

Furthermore, we reviewed the medical records of 15 available carriers of KP variants and their family members. We identified three tumor-associated variant carriers with a family history of breast-cancer-related diseases (Table 1). One of those patients was a female carrier of *BRCA1* KP variants (familial breast/ovarian cancer, MIM 604370), whose mother and maternal grandmother were diagnosed with benign breast nodules. The other two individuals both carried truncated *BRCA2* (familial breast/ovarian cancer, MIM 612555). One of them was a boy whose great-grandmother died of breast cancer, while the other was a girl whose mother was diagnosed with mammary hyperplasia. Additionally, we also identified two adolescents with a family history of cardiac diseases among those carrying variants associated with cardiovascular diseases (Table 1). One was a carrier of a *MYH7* (familial hypertrophic cardiomyopathy, MIM 192600) missense variant, whose grandfather was diagnosed with hypertrophic cardiomyopathy. The other was a carrier of the *TTN* (dilated cardiomyopathy, MIM 604145) truncation mutation, whose grandfather died of myocardial infarction and whose great-grandfather died of cardiomyopathy. The clinical manifestations of the affected relatives might be associated with the SFs variants, which were potentially shared between the carriers and their relatives. However, further follow-up of those carriers with family history was limited, which hindered us from obtaining detailed genomic data of their affected relatives.

In addition, we identified four individuals who exhibited additional cardiovascular phenotypes among those carrying cardiovascular-related KP variants (Table 1). Those findings might expand the phenotypic spectrums of *KCNQ1*, *SCN5A*, *PKP2*, and *TTN* (Table 1). For instance, *KCNQ1* (Long QT syndrome type 1, MIM 192500) and *SCN5A* (Long QT syndrome 3/Brugada syndrome MIM 603830/601144) have been associated with Long QT syndrome and sudden death. In our cohort, patient AIS18008000102 was a boy carrying a *BRCA1* nonsense variant and a *KCNQ1* splice region variant simultaneously. *KCNQ1* is rarely associated with cardiac structural defects, although this patient presented with atrial septal defect. Patient CSS16156400102 was a girl who carried a *SCN5A* splice region variant. Likewise, *SCN5A* is rarely associated with cardiac structural defects, although he was diagnosed with mitral valve dilatation. Neither patient AIS18008000102 nor CSS16156400102 had a family history of sudden death or severe cardiac diseases. We hence speculate that cardiac structural defects might expand the phenotypic spectrums of *KCNQ1* and *SCN5A*. Further clinical studies are needed to confirm our speculation.

Apart from the nine individuals with relevant family history or expanded phenotypes mentioned above, the remaining six individuals carrying KP variants were found to be free of significant clinical abnormalities. No abnormal phenotypes related to the KP variants were observed in those six individuals and their family members. 

## 4. Discussion

In this study, we screened for secondary findings variants in a heterozygous cohort according to the recommendation of the ACMG SF v3.0 list. We first analyzed the exome data of 2987 patients and interpreted the variants according to the ACMG guidelines. As a result, we identified 63 KP variants reported in the ClinVar or HGMD databases and 96 EP variants that led to truncated proteins. A further review of accessible medical records revealed the positive family history of five carriers of SFs variants. Expanded cardiovascular phenotypes were also observed in three cardiovascular-related SFs variant carriers, which potentially indicated the predisposition of SFs gene-associated diseases.

Apart from the main findings mentioned before, we also identified 37 heterozygous variants in six genes (*ATP7B*, *BTD*, *GAA*, *CASQ*, *MUTYH*, *TRDN*) associated with phenotypes inherited in an autosomal recessive model (Supplementary Table S1). Those variants were detected in 47 individuals, which accounted for 1.6% of the whole cohort. However, there is a lack of genetic evidence for pathogenic roles of those variants. Although those heterozygous variants did not meet the reporting criteria of SFs variants, it was still important to inform patients of the disease risks in their offspring. Further genetic counseling and early intervention could help improve affected children’s life qualities. 

In our study, SFs variants were detected and reported in 5.3% of individuals, which was inconsistent with other former reports (e.g., 2.3% in the Qatar population, 2.1% in the Turkish population, 2.85% in the Chinese population, 2.46% in the Korean population). Such differences could be mainly attributed to the expanded ACMG gene list we used for SFs variants screening, as the medically actionable variants of newly added SFs genes accounted for 28.3% (45/159) of the SFs variants in our cohort. Given the fact that the ACMG list of 73 genes was lately published last year, most of the existing SFs variants reported were based on the unrenewed gene list consisting of 59 genes. We also suggest that it might be necessary to re-evaluate the frequency of SFs variants in the East-Asian population based on the newest gene list, which served as a more comprehensive basis for diagnosis and subsequently prevention and/or eventually precise treatment of specific genetic disorders, if existing. 

Apart from distinct gene lists, the SFs variants screening results might also be affected by multiple factors in variant interpretation and filtering, including frequency threshold of variants, quality control criteria, interpretation tool (e.g., InterVar and Varsome), reference database (ClinVar and HGMD), and different filtering strategies, for instance, whether the expected pathogenic variants absent from reference databases were included in the SFs variants list. Those factors could explain the discrepancy between our study and other SFs reports based on the ACMG v3.0 SF gene list, which solely defined reportable SFs as P/LP variants reported in ClinVar, whereas our study included HGMD variants additionally [8]. This strict criterion led to a decrease in the SFs variant carrier rate in our cohort, from 5.3% to 3.4%. Hence, the filtering strategies we previously used could increase the sensitivity of SFs variants screening, while potentially decreasing the screening specificity. To report secondary findings variants more accurately and comprehensively, it is necessary for the research groups in different countries to cooperate and develop a uniform variant filtering strategy. 

In recent years, multiple studies have been performed to investigate the secondary findings and their impact in different ethnicities (Table 2). Compared to these studies, the frequency of SFs in our cohort (6.5% individuals carried SFs) was generally in line with that of the Lebanese population and mixed populations reported by Lawrence [7,19], but slightly higher than that of the Korean, Turkish, Qatari, Chinese, and Vietnamese mixed populations [9,20,21]. The variation of SFs frequency in different studies could be attributed to differences in genetic background. For example, the rate of pathogenic or likely pathogenic *ATP7B* variants in different populations ranges from 0.7% to 3.1% [6,22]. Additionally, differences in methodology might also affect the frequency of SFs. Chetruengchai et al. defined reportable SFs as pathogenic or likely pathogenic variants regardless of variants’ annotation in HGMD and ClinVar [8]. Elfatih et al. defined reportable SFs as LoF variants, HGMD (DM), and ClinVar (P/LP) variants, of which the methodology is consistent with our study [9].

We also reviewed some KP variants carriers, of which the medical records were accessible. We identified eight patients with *BRCA1* or *BRCA2* KP variants. A family history of mammary diseases including breast cancer was reported in three of them, accounting for 37.5% of the *BRCA* KP variant carriers. This result indicated high risks of specific diseases (and/or susceptibility) in the carriers with corresponding SFs gene variants. In addition, we also evaluated the clinical phenotypes unrelated to the primary diagnosis of those KP variants carriers. We observed phenotype expansions in the patients carrying KP variants associated with cardiovascular diseases. Phenotype expansions pose challenges to clinician in diagnosing genetic disorders, which highlights the importance of comprehensive genetic and clinical analysis.

In conclusion, this study was based on the latest ACMG SFs v3.0 gene list and focused on the prevalence of SFs variants in the Chinese population. We have identified SFs variants in 5.3% of the patients with unrelated primary clinical diagnoses, indicating a potential risk of developing severe genetic diseases in those individuals. Further follow-up and surveillance might promote the early diagnosis and precise treatment of specific human diseases.

## Figures and Tables

**Table 1:** Clinical characteristics of patients with follow-up.

Gene	Variant Name	Nucleotide Change	Protein Change	Carriers	Clinical Characteristics
*BRCA1*	chr17_41244976_G_A	c.2572C > T	p.Gln858Ter	CSS16261000102	Mother dx benign breast tumor; maternal grandmother dx benign breast tumor
*BRCA2*	chr13_32914982_C_T	c.6490C > T	p.Gln2164Ter	AIS18008000102	Great-grandmother dx breast cancer
*BRCA2*	chr13_32929364_CA_C	c.7379del	p.Asn2460ThrfsTer7	AIS18004900102	Mother dx hyperplasia of mammary gland
*MYH7*	chr14_23896019_G_A	c.2011C > T	p.Arg671Cys	OSS17011000102	Grandfather dx hypertrophic cardiomyopathy
*TTN*	chr2_179505980_T_A	c.40621A > T	p.Lys13541Ter	CSS16210400102	Grandfather dx myocardial infarction; great-grandfather dx cardiomyopathy
*KCNQ1*	chr11_2594217_G_T	c.921 + 1G > T	p.?	AIS18008000102	Carriers dx atrial septal defect
*SCN5A*	chr3_38662333_C_T	c.611 + 1G > A	p.?	CSS16156400102	Carriers dx mitral valve abnormality
*PKP2*	chr12_32949111_G_T	c.2421C > A	p.Tyr807Ter	OSS17011300102	Father dx hypertrophic cardiomyopathy combined with tricuspid valve abnormality
*TTN*	chr2_179454531_G_A	c.61921C > T	p.Arg20641Ter	AIS19013300102	Carriers dx mitral valve abnormality

We have followed up the carriers and their family members and recorded their clinical characteristics. The phenotypes potentially related to the SFs gene variants were exhibited in this table. Dx: diagnosis.

**Table 2:** Comparison of percentage with secondary findings in different studies.

Study	Sample Size	Ethnicity	Genes Included	Frequency with SFs (%)
**Kwak et al., 2017** [20]	1303	Korean	ACMG 56 (v1)	2.46
**Lawrence et al., 2014** [19]	543	Mixed	ACMG 56 (v1)	8.8
**Tang et al., 2018** [21]	954	Chinese and Vietnamese	ACMG 59 (v2)	2.50
**Yamaguchi-Kabata et al., 2018** [23]	2049	Japanese	autosomal genes (v2) in ACMG 59	21
**Nadine et al., 2020** [7]	280	Lebanese	ACMG 59 (v2)	6
**Ersa et al., 2021** [10]	622	Turkish	ACMG 59 (v2)	2.1
**Amal et al., 2021** [9]	6045	Qatari	ACMG 59 (v2)	2.3
**Wanna et al., 2021** [8]	1559	Thai	ACMG 73 (v3)	11.9
**This study**	2987	Chinese	ACMG 73 (v3)	5.3

SFs, secondary findings.

## Data Availability

The datasets generated and/or analyzed during the current study are available from the corresponding authors upon reasonable request.

## Supplementary Materials

The following supporting information can be downloaded at: https://www.mdpi.com/article/10.3390/jpm12091503/s1, Supplementary Table S1: Identified variants in this study.
